# The Prevention and Treatment of Prolapse

**Published:** 1939

**Authors:** H. J. Drew Smythe

**Affiliations:** Professor of Obstetrics, University of Bristol; Honorary Obstetrician, Bristol General Hospital


### THE PREVENTION AND TREATMENT OF PROLAPSE.

BY

H. J. Drew Smythe, M.D., M.S., F.R.C.S., F.R.C.O.G.,

*Professor of Obstetrics, University of Bristol;*
*Honorary Obstetrician, Bristol General Hospital.*

---

The Medical Practitioner has often been accused of causing, by bad midwifery, the majority of cases of prolapse ; the Obstetrician, of being also a gynæcologist in order to repair his mistakes. A certain number of cases of prolapse are the result of dealing with obstetrical difficulties, but the great majority will occur however careful the delivery. Women who in the non-pregnant state have lax abdominal muscles have lax muscles of the pelvic floor, and are therefore liable to prolapse even after a first confinement. The visceroptotic woman, who has frequently a congenital retroversion, and the obese, whose muscles are weakened by deposits of fat, are both liable to prolapse. Coughing and straining due to chronic bronchitis and similar disorders are often direct causes of prolapse.

There are two points in the delivery of the child which I should like to impress upon you as frequent causes of prolapse which can be avoided. The first is the premature application of forceps, namely before full dilatation of the cervix. When the cervix is fully dilated, the bladder, the cardinal ligaments and supporting tissues of the bladder are drawn up into the abdomen out of the way of the descending head and offer no resistance. If the cervix is not fully dilated these tissues are drawn down in front of the head and in consequence the supports are unduly stretched and torn. The result of this is uterine descent and cystocele, quite apart from the possible production of a cervical tear. Therefore, do not put on forceps until the cervix is fully dilated, unless maternal or fœtal distress demand interference.

The second point is, do not leave the head too long on the perineum if it is not advancing. The result of prolonged pressure is separation and stretching of the levator ani and superficial perineal muscles, which may never return to normal, and a rectocele is produced. You may as the result of holding the head on the perineum for a long time manage to keep the perineum intact, but in spite of this a rectocele forms later. It is rare to see a severe degree of prolapse with a complete perineal tear, whereas many cases of procidentia uteri and rectocele occur with an intact perineum. My advice is to do an episiotomy and deliver the head in all cases of undue delay on the perineum. The episiotomy must be accurately sutured at the end of labour.

Retroversion is a frequent complication of the puerperium and is usually due to subinvolution, or a previous congenital or acquired retroversion. Retroversion occurring during the early months of pregnancy will recur almost certainly during the puerperium. Sudden disappearance of the fundus on abdominal examination usually indicates a retroversion. Every patient should be examined vaginally about the tenth to twelfth day of the puerperium for this displacement. If retroversion is found, the displacement should be corrected and a pessary of the Hodge type inserted. The pessary should be worn for approximately three months. Long-standing retroversion will never be cured by a Hodge pessary. In the majority of cases retroversion causes no symptoms ; it is the complications of retroversion which cause symptoms. If symptoms are present, then operation is indicated, namely shortening of the round ligaments.

In considering the cases of uterine descent, cystocele and rectocele require appropriate vaginal repair. Ring pessaries should only be advised in cases of minor degree during the child-bearing period. All other cases of prolapse should be treated by operation. In the major degrees of prolapse the most satisfactory is the Fothergill or Manchester operation, in which the uterine ligaments are exposed by amputation of the cervix. These ligaments are shortened in the reconstruction of the cervix, and the anterior and posterior vaginal walls are repaired as in the older operations. In a certain number of cases in which the lesion is mainly a large cystocele in a woman past the menopause, with very little descent of the uterus, the interposition operation is of great value. In this the body of the uterus is interposed between the anterior vaginal wall and the bladder and forms an excellent support. The uterus must be of such a size that it will be sufficient to fill the gap. A senile uterus is of very little use.

In older women, where the above-mentioned operations are contra-indicated, Le Forte's operation may be employed. This consists in uniting the anterior and posterior vaginal walls from just below the cervix to the vulval orifice. Two lateral channels are left on either side for the escape of discharge. No woman should be advised to go through life with a prolapse or pessary when operation offers such a high rate of cure.

